# Retraction of the dissolution front in natural porous media

**DOI:** 10.1038/s41598-018-23823-3

**Published:** 2018-04-09

**Authors:** Y. Yang, S. Bruns, M. Rogowska, S. S. Hakim, J. U. Hammel, S. L. S. Stipp, H. O. Sørensen

**Affiliations:** 1 Nano-Science Center, Department of Chemistry, University of Copenhagen, Universitetsparken 5, DK-2100 Copenhagen, Denmark; 2 Helmholtz-Zentrum Geesthacht, Max-Planck-Straße 1, 51502 Geesthacht, Germany

## Abstract

The dissolution of porous materials in a flow field controls the fluid pathways through rocks and soils and shapes the morphology of landscapes. Identifying the dissolution front, the interface between the reactive and the unreactive volumes in a dissolving medium, is a prerequisite for describing dissolution-induced structure emergence and transformation. Despite its fundamental importance, the report on the dynamics of a dissolution front in an evolving natural microstructure is scarce. Here we show an unexpected, spontaneous migration of the dissolution front *against* the flow direction. This retraction stems from infiltration instability induced surface generation, which leads to an increase in reactive surface area when a porous medium dissolves in an imposing flow field. There is very good agreement between observations made with *in situ*, X-ray tomography and model predictions. Both show that the value of reactive surface area reflects a balance between flow-dependent surface generation and destruction, i.e. the “dry” geometric surface area of a porous material, measured without a flow field, is not necessarily the upper limit of its reactive surface area when in contact with reactive flow. This understanding also contributes to reconciling the discrepancies between field and laboratory derived solid-fluid reaction kinetics.

## Introduction

In a reacting porous medium, a reaction front is an isosurface over which the chemical affinity of a reaction reduces to zero in the velocity direction. This isosurface separates the reacting surface from the remaining geometric surface and is the ideal boundary of the region of interest (ROI), for measuring the kinetics of water-rock interactions. At any given instant, the inherent heterogeneities of natural porous materials that are not encompassed by this isosurface do not contribute to the chemical reaction. Therefore, the ability to track an evolving reaction front can greatly simplify the analysis of many geochemical processes by allowing unambiguous identification of the temporal ROI.

The dissolution front is the reaction front of a solid dissolution reaction. In a dissolving medium with a pressure driven flow field, there is a positive feedback between the mineral dissolution rate and the local permeability (Yang, Y. *et al.*, Submitted, 2017)^1,2^. This feedback leads to morphological instability of the migrating dissolution front, referred to as reactive infiltration instability (RII)^3,4^. RII is fundamental to many geological self-organisation phenomena^4,5^. However, the behaviour of a dissolution front in the presence of the RII in natural microstructures remains elusive^6–10^, presumably because of the technical challenge of observing it experimentally^11–14^ and effectively incorporating parameters to describe the inherent heterogeneities of porous media into numerical simulations^3,15–21^. A comprehensive overview of the subject can be found in Szymczak *et al*.^3,19^ and references therein. A formulation of the problem from the perspective of a feedback loop consisting of flow field, reactivity field and solid-fluid conversion is presented in (Yang, Y. *et al*., Submitted, 2017).

Here we show an unexpected, spontaneous migration of the dissolution front *against* the pressure gradient in a flow field. Different from previously reported retraction of reactants in binary geometry^22^, the front retraction we show stems from the tendency of infiltration instability to amplify heterogeneity and to enhance local microstructural contrast. This instability results in the generation of reactive surface that does not rely on the displacement of fluid-solid interface. There is very good agreement between *in situ* observation and numerical simulation, both suggest that the front retraction is applicable from the perspective of a complete flow domain, i.e. the result is not derived from a partial view of an evolving microstructure and reflects the competition and merging of multiple pores developing in parallel.

## Results

### Surface generation by infiltration instability

Given a flow field, the position of the dissolution front along any streamline can be determined using a plug flow approximation:

1 $${\int }_{p{H}_{0}}^{p{H}_{eq}}\frac{d[{{\rm{H}}}^{+}]}{{r}_{diss}}={\int }_{{\bf{s}}}\frac{A}{{\bf{v}}}ds,$$

where [H^+^] represents the hydronium ion concentration (mol·m^−3^), pH_0_ and pH_*eq*_, represent the initial and equilibrium states of the rock dissolving fluid (pH used as a master variable), *r*_*diss*_ represents the mineral dissolution rate (mol·m^−2^·s^−1^), which typically depends on pH and the mineral saturation index (SI); **v** represents the fluid velocity and **s**, the streamline as the integral path. *A* represents the specific surface area (i.e. the surface area per unit volume, m^2^/m^3^). In the numerator, surface area is defined as the spatial change of material density (see Methods for an example of surface area calculation) and two concepts of surface area are used in this study. Geometric surface area (GSA) is the surface area within a sample or a simulation domain and is independent of the imposed flow field. Reactive surface area (RSA) is the portion of GSA on which the dissolution reaction occurs, i.e. chemical conversion is not zero. We use the word *area* when a quantity is referred to, i.e. GSA and RSA refer to the measurable amounts of geometric and reactive surfaces, respectively. Equation 1 relates the chemical potential of the fluid (left side, LS) to the physical properties of the medium (right side, RS). The LS is constant for a specific reaction and initial fluid composition. The length of ***s*** needed for the RS to equal the LS varies greatly along different flow paths, owing to the heterogeneous spatial distribution of flow velocity and the geometric surface^23^. In a chalk sample with a steady state flow field, variation in the physical properties (represented by the RS) of as much as 6 orders of magnitude has been observed (Yang, Y. *et al*., Submitted, 2017). The dissolution front, as an isosurface, consists of all the points on each and every ***s*** that satisfies Equation 1 so is a morphologically complicated surface in 3D (Fig. 1a). Greater specific surface area (*A*) or longer residence time (s/v) yields shorter ***s***.

**Figure 1:**
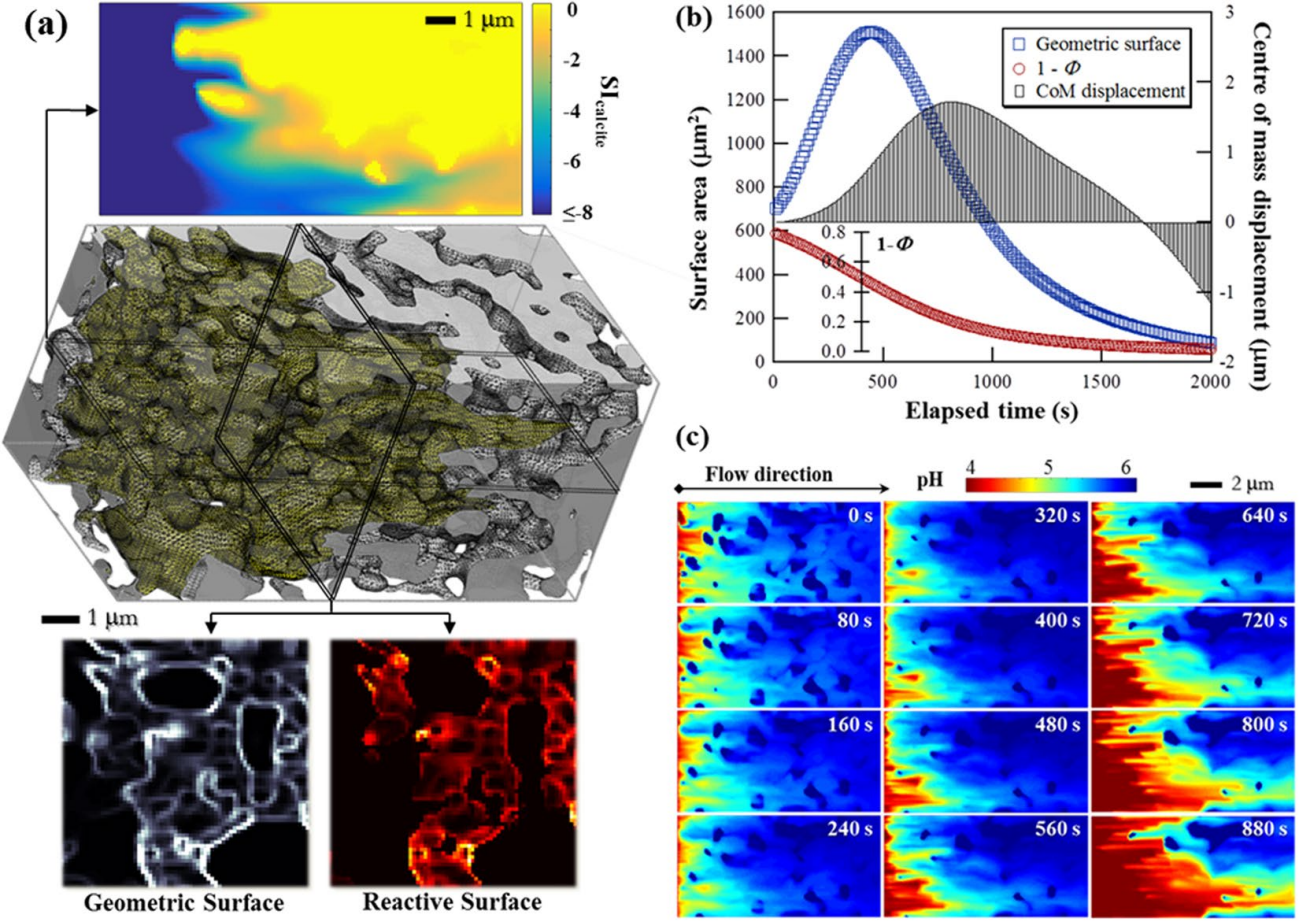
Reactive transport simulation from a greyscale, nanoCT dataset of natural chalk. (**a**) A perspective view of the initial microstructure (7 × 7 × 14 μm^3^). The instantaneous reaction front is imposed on the structure as a yellow isosurface. Fluid flows from left to right. Above the structure is a cross section of the distribution of calcite saturation index in the initial flow field. The dissolution front separates the yellow region (SI_calcite_ = 0) from the rest of the domain. At the bottom are images showing the instantaneous geometric and reactive surfaces of the same cross section. Only the geometric surface contained by the reaction front is reactive. (**b**) The evolution of geometric surface (blue squares) and solid content (1 − *Φ*) of the simulation domain (red circles). Also shown is the change in the centre of mass (columns), which reflects the distribution of solid along the flow axis. (**c**) The evolution of the pH distribution. The same axial cross section in (**a**) is used. The initial pH of the solution was 3.9. The migration of the dissolution front in the opposite direction of the flow was observed from *t* = 0 to 400 s.

Infiltration instability amplifies the transport heterogeneities in a porous medium by channelling the more reactive fluid toward the more permeable flow paths^4^. For example, local permeability differences, often stemming from the local variations of material density, can be amplified by RII^24^. The solid-pore interfaces in porous media are defined by sharp spatial change of material density^25^. The pore surfaces are also the sites of fluid-solid interaction. Therefore, RII can increase the internal surface of a porous medium. In a simulation of a 7 × 7 × 14 μm^3^ volume of real chalk (Fig. 1a), the GSA increased from 7.0 × 10^2^ μm^2^ to 1.5 × 10^3^ μm^2^ in 450 s (Fig. 1b). The simulated percolation used an acidic solution (ultrapure water, equilibrated with 1 bar CO_2_ at 25 °C; pH = 3.9) and a constant volumetric flowrate (5 μm^3^/min). The GSA started decreasing after 450 s because of solid depletion. The same figure also shows that the non-monotonic microstructure evolution cannot be seen from the overall mass balance. The solid residual, indicated by 1 – *Φ* (overall porosity), decreased monotonically while the centre of mass (CoM) in the axial direction changed with time. First it moved toward the fluid outlet (positive displacement) because of preferential upstream dissolution but then later, it moved backward because solid near the fluid entrance did not react, bypassed by the channelled fluid.

The flowing fluid approaches equilibrium rapidly because of the increased surface area. As a result, the dissolution front moves against the flow field toward the fluid entrance. This is demonstrated by the temporal pH mapping of the simulation domain (cross sections shown in Fig. 1c). pH varied between 3.9 (initial) and 6.1 (equilibrium) and the dissolution front retraction took place between *t* = 0 (the instant when the microstructure was allowed to change in the established flow and concentration fields) and *t* = 400 s. During this period, the region of the warmer colours (Fig. 1c; the more reactive subvolume of the domain) shrank toward the left boundary because of the constantly increasing *A* on the RS of Eq. 1. A film is provided in Movie 1.

Figure 2a shows the dissolution rate as a function of the axial position, averaged over the cross section. With the initial microstructure, the dissolution rate decreased to 10^−8^ mol·m^−2^·s^−1^ within 3.5 μm. After 200 seconds however, this distance moved, unexpectedly, opposite to the flow direction by 1.5 μm. At *t* = 400 s, the position had retracted another 0.4 μm. This decrease in the retraction speed resulted from solid depletion near the fluid entrance (Fig. 2b). At *t* = 600 s, the reaction front advanced in the flow direction to 2.7 μm, indicating that the RII induced surface generation had been balanced by the limited solid availability. This is in accordance with the observed change in the surface area distribution along the axial direction (Fig. 2c). At *t* = 600 s, the GSA near the inlet was lower than in the previous time steps but at the same time, the GSA between 8 and 12 μm in the axial direction was still increasing. If we consider the portion of GSA on which the reactant consumption is not zero as the reactive surface area, it is expected that in a porous medium that is infinitely long in the flow direction, the reactive surface area at any instant reflects the dynamic balancing between the RII-induced surface generation and the surface destruction caused by solid depletion. This means that, the reactive surface area of a porous medium in a flow field is not the inherent geometric surface and not necessarily less than the “dry” GSA, that is, the surface area measured without imposed flow. Hence, using a “dry” GSA as the upper limit to estimate reactive surface can introduce uncertainties in the analysis or simulation of water-rock interactions in a flow field. This is an important point.

**Figure 2:**
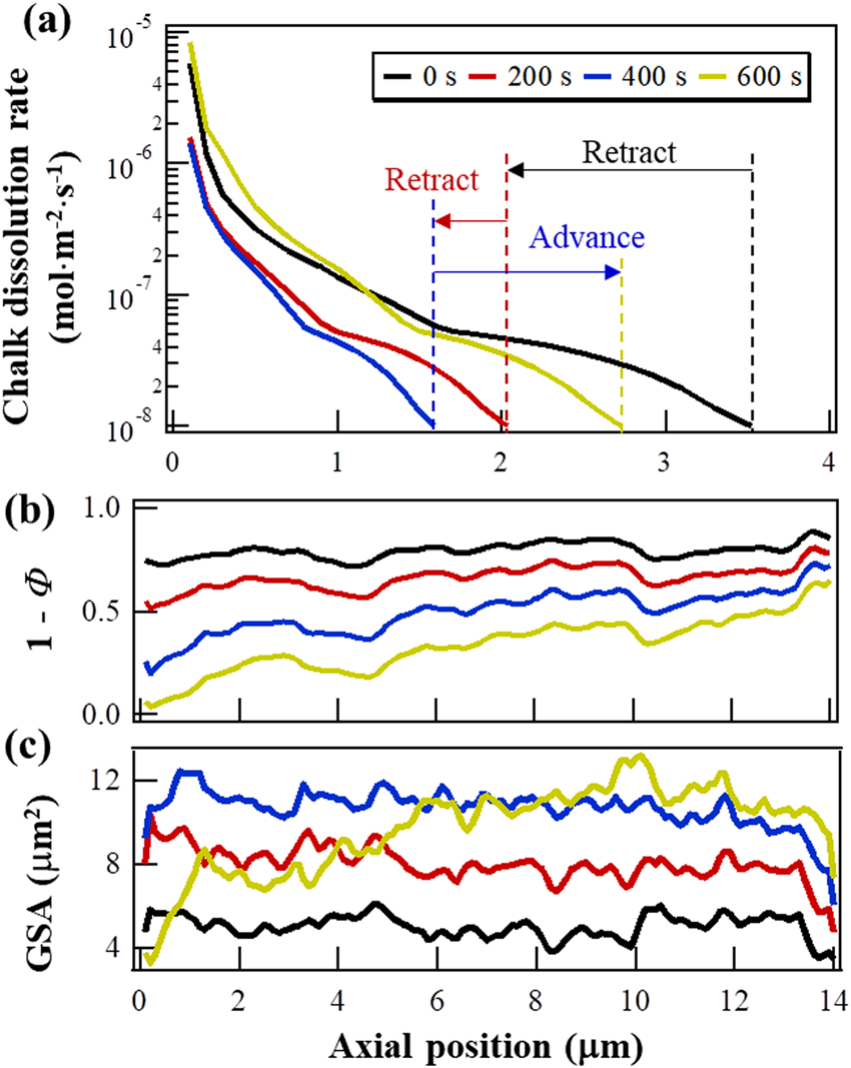
Evolution of the axial heterogeneities in the numerical simulation. The origin of the *x* axis is the fluid inlet. (**a**) Distribution of chalk dissolution rate along the flow direction. The rate dependence on the cumulative surface is computed based on a related work (Yang, Y. *et al*., Submitted, 2017). (**b**) Distribution of solid material along the axial direction, whereas *Φ* represents the porosity averaged over the plane perpendicular to the flow. (**c**) Distribution of geometric surface area (GSA) along the axial direction.

### Receding dissolution front recorded by X-ray imaging

We used *in situ* X-ray microtomography (μCT) to monitor the dynamics of the microstructural evolution discussed above. A cylindrical chalk sample (ø × L = 0.9 × 2 mm) was used as a fast reacting model rock. The percolation fluid (ultrapure deionised water equilibrated with 8 bar CO_2_ at 50 °C) and slow flow rate (0.016 ml/min) were chosen such that the dissolution front would be contained within the field of view (FOV) accessible with μCT. The density of the porous material was reflected by the local, X-ray, linear absorption coefficient of the sample^26^. The sample’s internal geometric surface was indicated by the spatial variations in porosity^25^. Both quantities were measured from the intensity units of the reconstructed greyscale images.

Dissolution was recorded continuously over a period of 88 hours. Figure 3a shows a monotonic decrease in sample density, with a preferred dissolution region near the fluid entrance. Figure 3b shows the evolution of the geometric surface along the flow direction. The values are the norms of the 3D intensity gradient vectors integrated over all the corresponding planes. A temporal increase of GSA was evident at *t* = 28.5 h (red). Figure 3c shows the μCT images corresponding to the cross section at 650 μm from the fluid inlet (indicated by the grey dashed line in Fig. 3a and b). A Sobel-Feldman operator was applied to the images to highlight the geometric surfaces^27^. Comparison of the images from *t* = 0 and 28.5 h indicates that the amplification of the pre-existing heterogeneities contributed considerably to the increase in surface area – a phenomenon characteristic of infiltration instability^2^. Fig. 3d shows the evolution of the sample density (red circles) and the geometric surface (blue squares) averaged over the whole FOV (1 × 1 × 2 mm^3^). Also shown is the displacement of the centre of mass in the axial direction determined from X-ray absorption (columns). The dynamics of these quantities agrees very well with the numerical simulation (Fig. 1b).

**Figure 3:**
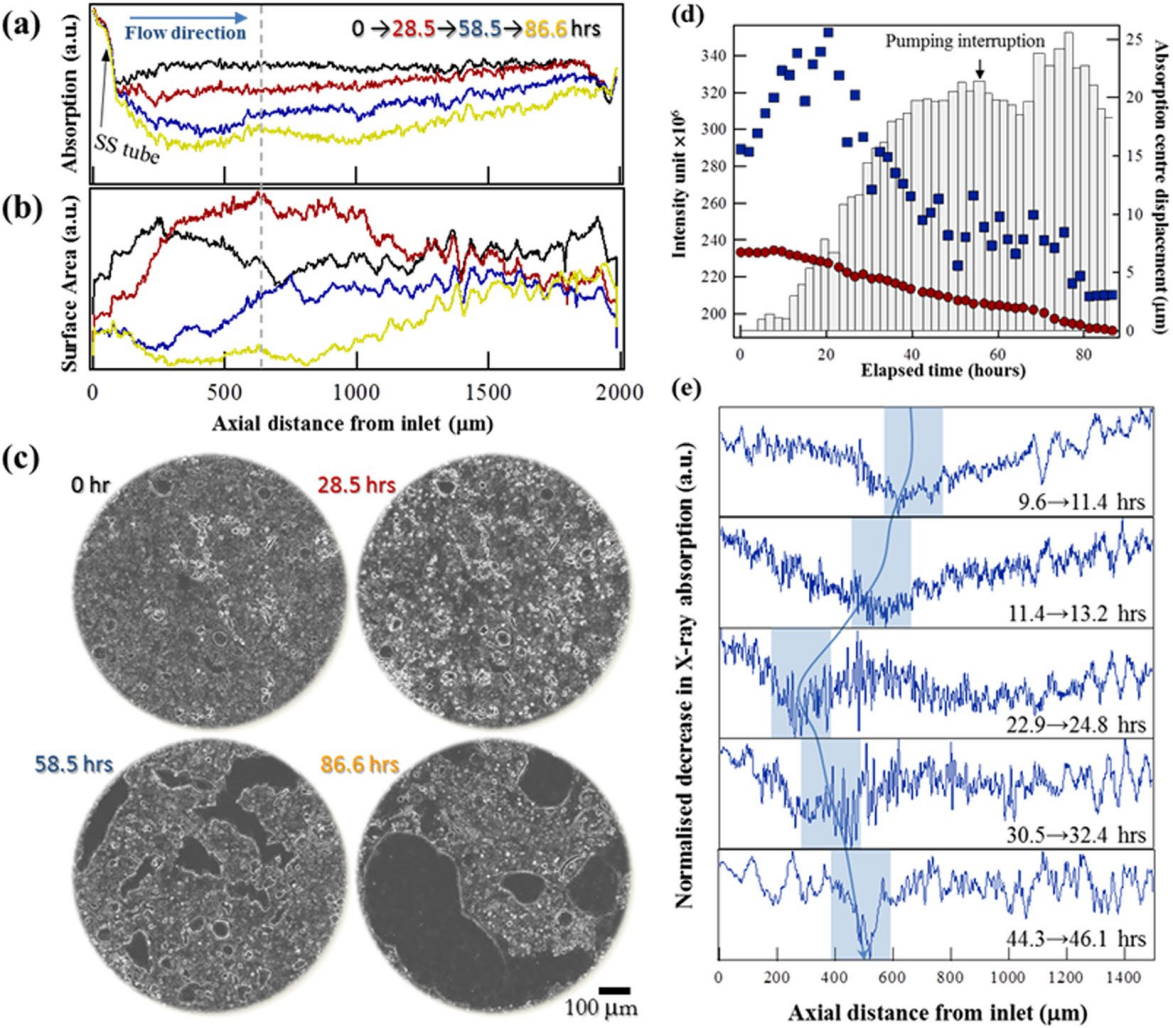
Evolution of chalk microstructure recorded by *in situ* X-ray tomography. (**a**) Axial distribution of X-ray absorption, which reflects the average density of the sample in the radial direction. The strong absorption near the fluid inlet was caused by a stainless steel (SS) tube (arrow). (**b**) Axial distribution of geometric surface area, calculated as the norms of the 3D intensity gradient vectors integrated over the radial plane. (**c**) Cross sections of the evolving microstructure, 650 μm away from the fluid inlet (the grey dashed line in **a** and **b**). The intensity contrast is highlighted using the Sobel-Feldman operator. The pre-existing structural heterogeneities were greatly enhanced during the first 28.5 hrs of the experiment. (**d**) Evolution of X-ray absorption (red circles), geometric surface (blue squares) and the displacement of centre of mass (columns, measured by X-ray absorption) of the complete field of view (FOV). There was an interruption of fluid pumping during the *in situ* measurement at about *t* = 60 h. (**e**) Decrease of X-ray absorption between time steps as a function of axial position. The shaded areas show the regions where significant solid was removed during sequential scans.

In the 4D dataset (the time series of the evolving 3D structure), the intensity at each time step was subtracted from that of the following step so the difference reflected a spatial distribution of the solid removal rate. Figure 3e shows this distribution in the axial direction for 5 pairs of consecutive time steps. The smaller the value, the more solid was removed. The shaded areas connected by the blue line show qualitatively the evolution of the most reactive portion of the sample. Retraction of the dissolution front toward the fluid inlet was observed in the first 3 time steps (pairs), in agreement with the increase of surface area shown in Fig. 3a and d. The subsequent advance of the shaded region resulted from solid depletion, which is directly observable from the image stacks (Movies 2 and 3).

## Discussion

The unexpected retraction of the dissolution front is different from the receding of reactant observable in an expanding pore as part of a flow field, although both are associated with an increase in the surface area as pore structure develops. The surface increase in this study is directly related to infiltration instability and to the enhancement of local porosity/permeability contrast resulting from this instability. In both the simulation and the *in situ* measurement, we have adopted a definition of surface area that does not rely on the displacement of solid-fluid interface. The complexity of a porous medium is typically characterized by a two point correlation function^25^. This correlation describes the probability of any two random points in a porous medium belonging to the same phase as a function of their distance. When calculating the specific surface area *A* of the medium, one calculates the first derivative of this probability density function as the distance approaches zero. The physical significance of this method is that the geometric surface in a porous material is defined as the amplitude and frequency of the spatial variations of material density. This is a definition applicable to both greyscale and binarised tomographic datasets. With this definition, surface area can increase in a greyscale dataset if the porosity difference between any neighbouring voxels is enhanced. This manner of surface increase is not possible in a binarised dataset because the contrast of porosity is fixed at 1 and the voxels are assigned to either solid or pore. Therefore, a mechanism that relies on the expansion of idealized (e.g. cylindrical), binary pore geometry to explain reactant retraction has to invoke the displacement of solid-fluid interface and cannot explain the surface area generation stemming from any locally enhanced structural difference.

Meanwhile, in both our simulation and *in situ* measurement, the enhanced local porosity contrast was the predominant reason for the increase of RSA. In the greyscale simulation, the solid-fluid interface was not tracked explicitly and no idealized/simplified geometry had been assumed for surface area calculation. Only after a predominant flow channel appeared was the mechanism of pore expansion applicable to the highly irregular shape of the channel. However, when this expansion began, the overall surface area had already started to decrease because of solid depletion. The expansion of pore dimension thus cannot explain the momentary increase of surface in our simulation. Similarly, in the *in situ* measurement, the surface area was defined by the intensity contrast of neighbouring voxels. Because of the very fine grains of chalk, the *in situ* μCT generally do not fully resolve the features and most of the voxels contain a mixture of void space and mineral and is therefore represented by a greyvalue. As is shown clearly in Fig. 3c, the boundaries of grains were significantly enhanced after 28.5 hours, before the disintegrated structure began to move. In other words, although the *in situ* μCT clearly showed an increase of surface area in the greyscale images (according to the adopted definition), no evidence showed that this increase was related to the expansion of any fully resolved, pre-existing pores. Instead, the enhancement of porosity contrast in the simulation and of the intensity contrast in the *in situ* measurement were both in concord with the inherent tendency of infiltration instability to amplify local structural differences.

Another factor that differentiates our results from previous reports is that our investigation – both simulation and experiment – covered the whole flow domain in a physically realistic microstructure, rather than focusing on a single pore. If the reactant retraction is derived, theoretically, from a single pore geometry with a special analytical form (e.g. cylindrical), it can be challenging to prove that similar phenomena can be expected for any other, most likely highly irregular, geometry in natural porous media. The focus on a single pore has also excluded the consideration of the competition and merging of pores developing in parallel. For example, one would not be surprised by seeing a retraction of reactant from a pore that is failing the competition to channelize reactive fluid. This retraction, however, is contributing to the advancement of the reaction front in other pores that can only be observed if the complete flow field is monitored.

Nevertheless, it is a great question to ask whether, within a fully resolved rock sample, the development of pores can always be perceived as an expansion of binary pore geometry on a sufficiently small scale. If so, the enhancement of density contrast near the reaction front as a result of infiltration instability loses its physical significance because material density is inherently an continuous (and hence, greyscale) property. An unambiguous answer to this question will rely on a fully resolved wormholing process, i.e. seeing through the development of pores without unclassified, grey voxels. This level of imaging capacity is currently beyond our reach. However, one could speculate a potential paradox in the binary preconception. If a rock sample is not perfusable, i.e. pores are not interconnected in the direction of the pressure gradient, one cannot observe competing wormhole development because the transport of reactants and products in the “dead” pores rely exclusively on diffusion and therefore, the positive coupling between advection and mineral dissolution does not form. If a sample is perfusable, wormholing becomes a pre-templated process where the many, pre-existing flow paths expand. In either case, no new, perfusable pore is created in the flow direction, contradicting our observation with the *in situ* imaging.

It is also of interest to put the results into the context of the conventional conceptual framework of the instability problem – that is, to define dissolution regimes (face dissolution, wormholing or uniform dissolution) based on visual “patterns” that relies on the relative size of the system dimension and the reactant penetration depth. No front retraction can be observed during face dissolution in the absence of infiltration instability. The prerequisites for face dissolution are that (1) the penetration length of the reactant is small compare to the field of view (FOV) and that (2) the migrating front is, to a large extent, stable. In face dissolution, the negative feedback, often stemming from both diffusion and the limited availability of solid, is strong and deteriorates the growth of perturbation. The dissolution front propagates as a plug flow and do not amplify heterogeneities on its pathway. In contrast, for uniform/homogeneous dissolution, the front retraction may or may not exist. This uncertainty is related to the fact that this dissolution regime (“uniform”) merely tells that the penetration length of the reactant is greater than the FOV. It does not contain the information whether instability exists within the FOV. The reaction “front” is a very special reaction rate isosurface, where the Gibbs free energy increases to 0 in the flow direction. In general, there is an infinite number of rate isosurfaces in the flow field, where the specified rate does not have to equal zero. Any of these isosurfaces is subject to similar stability analysis as is the reaction front. In a uniformly dissolving medium, if all rate isosurfaces are stable, then one would not observe any enhancement of heterogeneity leading to surface generation. The front retraction does not exist because of the absence of instability. On the other hand, if any of the rate isosurface is not stable, then instability exists and a retraction of the front is possible. It is worth emphasizing that whether instability exists or not does not rely on whether the FOV can capture the entire reactive subvolume, or whether the reaction front develops within the limited size of a system.

On a related note, the qualitatively similar observations across the few orders of magnitude are subject to a similar argument. There are two similarities between the experimental observation and the numerical simulation. First, a transient increase in surface area, leading to the receding of reaction front, was identified in both cases. Second, the subvolume of the system that showed an increase in surface area moved in the flow direction, both experimentally and in the numerical simulation. These similar behaviours are not specific to any length scale and are grounded on two common factors. First, the increase of surface area relies on a balance between surface generation and destruction. New surface is constantly generated given that there is a positive feedback between fluid flow and dissolution reaction. This positive feedback is scale invariant and relies only on the simultaneous presence of advection and reactants. Surface destruction is a natural consequence of solid depletion, which is subject to mass balancing and is not scale specific either. Second, the observation of the reactive subvolume migration in the flow direction relies on the control of the reactant penetration depth. This control was achieved by choosing the flow rate in the experiment so that the ratio between the reactant penetrated subvolume and the whole domain was similar to the same ratio in the numerical simulation, i.e. the flow rate in the simulation needed to be much smaller than that in the experiment so only a portion of the domain was reactive at any given moment. The same strategy of penetration control can be used on any length scale, given that the predominant mass transfer mechanisms are known. These two common factors resulted in the similar behaviours on the two length scales.

The migration of dissolution front opposite to the pressure gradient in the flow field suggests the potential need to re-evaluate former determinations of dissolution kinetics. Accurate quantification of the reactive surface area is associated to many important topics in water-rock interaction (*e*.*g*., the Gibbs free energy dependence of reaction rate)^28^. We have shown that the surface area of a porous material can be different with and without flowing fluid^2^. Perhaps more surprising is that, given the same rock sample, the RSA can be greater with a flow field, even when the sample is chemically homogeneous and when there is no precipitation of secondary phases. This contradicts the long held assumption that the geometric surface area measured *ex situ* is the upper limit for the reactive surface area, as the latter is subject to fluid accessibility. This difference may also have contributed to the repeated reporting of discrepancies between laboratory and field kinetic measurements^29^. In reality, in many geologic systems, flow is divergent, *i*.*e*., velocity decreases with distance from the fluid source. In such cases, from the cumulative surface perspective (RS of Eq. 1), the natural porous medium can be considered infinitely long in the flow direction and the balance between surface generation and removal determines the interface for fluid-rock interactions. To better quantify such complex dynamic processes, there is an urgent need to investigate the rate of RII-induced surface generation, the upper limit for the specific surface area reflected by the fractal dimension of geologic materials, as well as the rate of surface destruction, controlled by solid availability.

## Methods

### X-ray computerised tomography

A model environment for the numerical simulation was created using X-ray holotomography reconstructions^30^ of drill cuttings from North Sea Hod chalk. We used a sample that was roughly cylindrical, with diameter of ~500 µm. Data were collected at the ID22 beamline at the European Synchrotron Research Facility (ESRF) in Grenoble, France. We collected 1999 projections from a single dry scan at 29.5 keV, with an exposure of 500 ms and 360° rotation. The reconstruction had a voxel dimension of 100 nm^30^. For this study, the reconstructions were intensity aligned, compensated for ring artefacts, denoised with iterative nonlocal means denoising and sharpened by deconvolution. An estimate of voxel level porosity was acquired by interpolating from an intensity based Gaussian mixture model^31^.

Percolation experiments were made with outcrop Maastrichtian chalk collected near Aalborg, Denmark (Rørdal Quarry). The material contains primarily CaCO_3_ in the form of calcite, from fossilized coccolith and skeletal debris, with ~4% silica content. The average porosity is high, ~45%, permeability ranges between 3 and 5 mD and the BET surface was 7.31 m^2^/g^32^. We loaded our miniature version of a Hassler core holder (Yang, Y. *et al*., Submitted, 2017) with an Aalborg chalk sample machined into a cylinder of 900 µm diameter and ~2 mm length. To guide the flow of solvent, the chalk cylinder was mounted inside heat shrink tubing (3 M, Maplewood, MN, USA), placed between two stainless steel needles that served as fluid in- and outlets and sealed with epoxy resin prior to loading. The sample was confined in ultrapure water at 10 bar at the beginning of the experiment. The shell side containing the confining fluid was then sealed. A syringe pump (260D, Teledyne ISCO) was used to drive the ultrapure water (equilibrated with 8 bar CO_2_ at 50 °C) through the sample at 0.016 ml/min.

The dissolution process was monitored by continuous imaging using X-ray microtomography (μCT) at the P05 beamline of PETRA III at the Deutsches Elektronen-Synchrotron (DESY). The beamline is operated by the Helmholtz-Zentrum Geesthacht^33^. Over the course of 88 hours, 45 consecutive tomography datasets were collected, with beam energy of 28 keV. Each set consisted of 1200 projections exposed for 1050 ms over 180° rotation. The time resolution was ~110 min. The effective voxel size in the reconstructions was 2.66 µm after down sampling the 3056 × 3056 pixel projection data by a factor of two in both directions. Prior to reconstruction, the interface of the aluminium wall of the core holder to the confining water was tracked and matched with a reference sinogram to compensate for drift and distortion caused by a changing drag force from the tubing during sample rotation. Ring artefacts were suppressed by Fourier-wavelet destriping of the sinograms before reconstructing with the GridRec algorithm^34^. After reconstruction, we applied iterative non-local means denoising (through 4 cycles) to every time step of the 4D dataset, using a constant noise level estimate and we aligned the dataset spatially, with digital volume correlation using Pearson’s correlation coefficient as the quality metric. Details of the signal processing can be found in Bruns *et al*.^31,34^.

### Greyscale model and simulation

For numerical simulations, we built a reactor network model that treats each voxel as a mixed flow reactor (MFR) and its connection in its octagonal neighbourhood (i.e. 6 nearest neighbours) as an ensemble of plug flow reactors (PFR)^35^. When a flow field is imposed, the model allows the spatial distribution of voxel porosity to be related to the reactor properties (Yang, Y. *et al*., Submitted, 2017). The simulation domain contains 70 × 70 × 140 = 686,000 voxels, corresponding to a 7 × 7 × 14 μm^3^ volume of chalk sample. Within each 100 nm voxel, we assumed a linear dependence of the permeability and tortuosity on the voxel level porosity. This assumption means that the pressure drop in the PFR, modelled by Darcy’s law, uses a phenomenological coefficient in the form of a parabolic function of local porosity given by the geometric mean of the connected reactors. The flow field was then solved by the stabilized biconjugate gradient method under the assumption of incompressible flow and continuity.

The reactor model relates the reactant concentration with the mixing state in the reactor. Previous studies illustrated that the local conversion can be determined by the voxel level Damköhler number^36,37^. A 50% subvolume of each voxel was treated as an MFR. An expression of surface area within each PFR is retrieved from the norm of the porosity gradient vector multiplied by the 2^nd^ power of the voxel dimension. The geometric surface area of each voxel is then evaluated by summation over all neighbouring voxels. For example, if an empty voxel (porosity = 1) is surrounded by six solid voxels (porosity = 0), then its geometric surface area is *l*^2^ × 6 × |1 − 0|/2, where *l* is the voxel dimension (100 nm) and the divisor 2 appears because each interface is share by two neighbouring voxels. Half of this geometric surface area is assigned to the MFR (1.5*l*^2^) while the other half is assigned to the PFRs (each gets 0.25*l*^2^). The reactive surface area is then calculated according to the chemical conversion in each MFR and PFR. In this example, if Ca^2+^ is released in the MFR and in two of the six PFRs, the reactive surface area of the voxel is 1.5*l*^2^ + 0.25*l*^2^ × 2, i.e. 20,000 nm^2^. The geometric surfaces in the other four PFRs are ignored.

The dependence of chalk dissolution rate on aqueous calcium concentration was obtained by (1) calculating the far-from-equilibrium rate using the rate law proposed by Plummer and Busenberg^38^ and (2) measuring the evolution of aqueous calcium concentration in a closed free-drift system with dissolving chalk powder. We then solved the Fredholm equation of the 2nd type, an integral differential equation, to delineate the evolution of surface area from that of dissolution rate. The derivation can be found in (Yang, Y. *et al*., Submitted, 2017). This rate profile was then mapped to the LS of Equation 1 and reflects the decrease of fluid reactivity as the cumulative surface along a flow path increases. If the reciprocal of the LS of Equation 1 is used as the pseudo-first order rate constant, it yields a value of 3.15 × 10^−7^ m/s and the macroscopic Damköhler number (Da) for the simulation is ~10. The physical significance of this Da is that the ratio between the volume of the system (the simulation domain) to the reactive subvolume of the system (penetrated by reactive fluid, encompassed by the reaction front isosurface) is 10:1 at any instant. pH and the saturation index in each voxel (Fig. 1) were calculated with PHREEQC^39^, using the total aqueous calcium concentration as input. The concentration field was then solved iteratively, based on the flow field, until the Frobenius norm of the difference between two consecutive iterations was less than 10^−6^. Each iteration was initialized with the inlet concentration of the previous iteration. The simulation only considers transport of aqueous calcium, i.e. pH and saturation index in each reactor were calculated assuming calcite as the only available source of calcium in a closed compartment. The voxel porosity was updated using mass balance after the chemical conversion in each reactor had been determined. The time step of the simulation was chosen adaptively, such that the overall porosity change would not exceed 0.01 in each step. Further details can be found in (Yang, Y. *et al.*, Submitted, 2017).

### Data Availability

All data needed to evaluate the conclusions in the paper are presented. Additional data related to this paper may be requested from the authors by emailing yiyang@nano.ku.dk.

## Electronic supplementary material

Movie 1

Movie 2

Movie 3
