# Supplementary figures and images for: Retraction of the dissolution front in natural porous media
Source: Sci Rep. 2018 Apr 9;8:5693. doi: 10.1038/s41598-018-23823-3 (PMC5890250; doi:10.1038/s41598-018-23823-3)

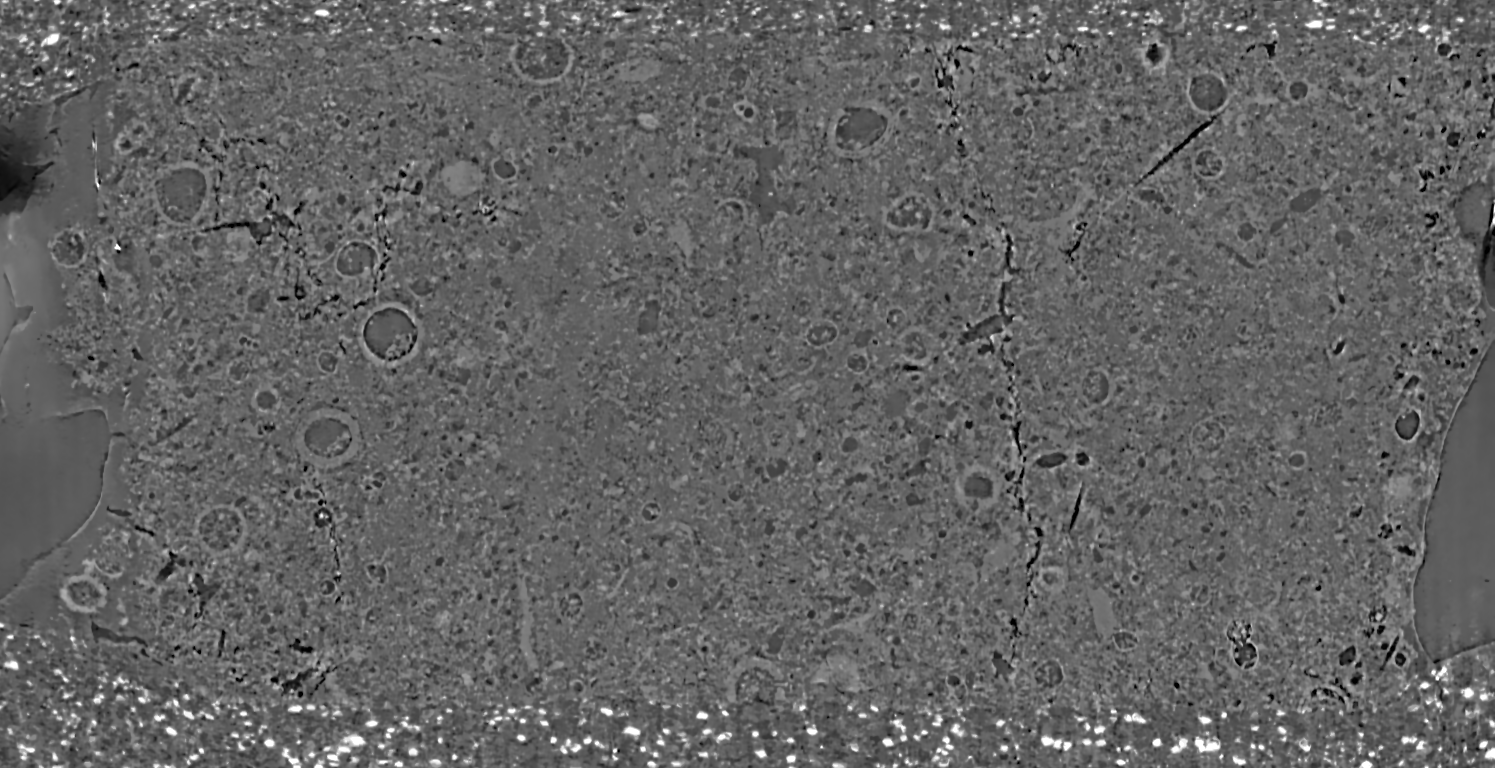

Supplement: Supplementary file 2 — Movie 2 [file 41598_2018_23823_MOESM2_ESM.gif]

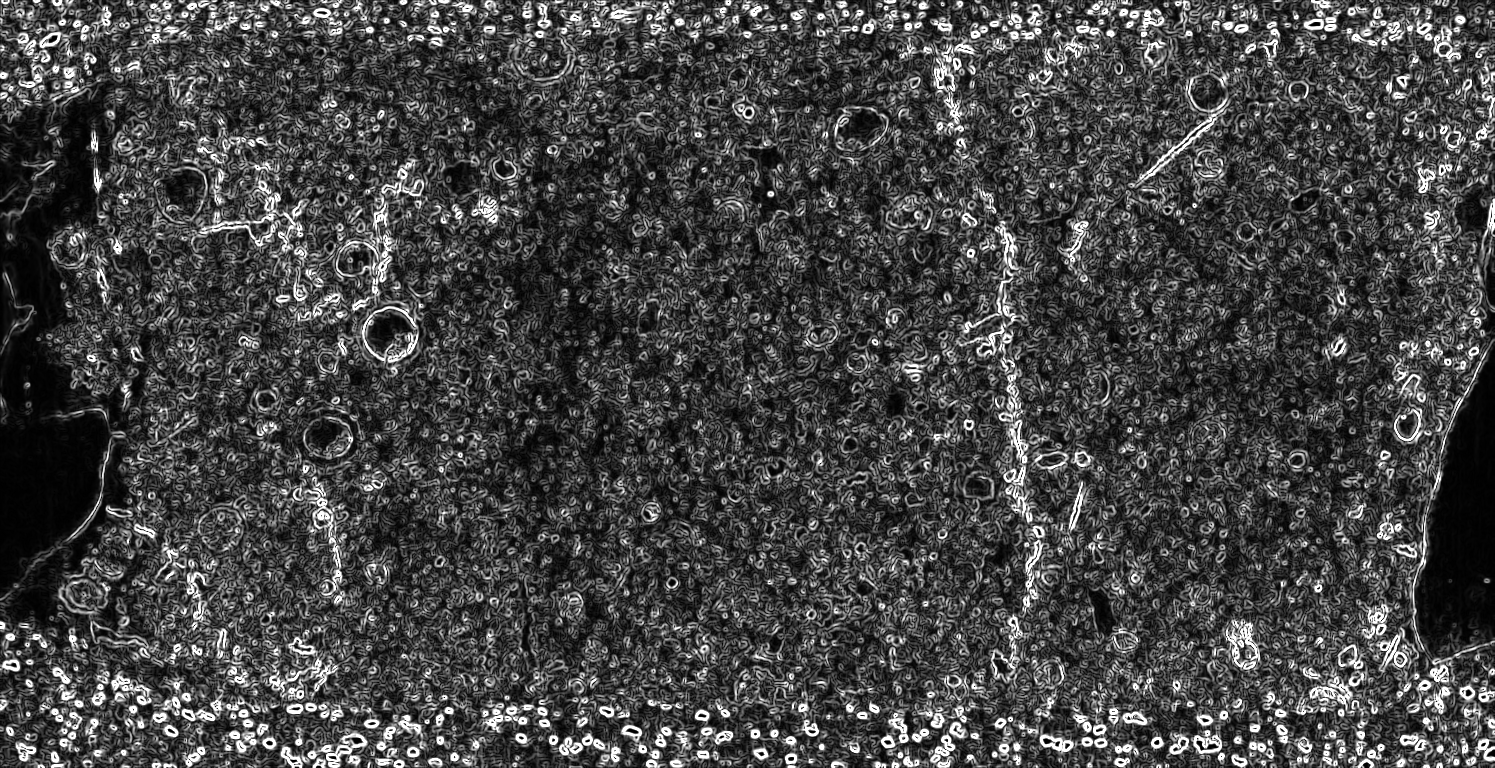

Supplement: Supplementary file 3 — Movie 3 [file 41598_2018_23823_MOESM3_ESM.gif]
